# Supplementary figures and images for: Idiosyncratic evolvability among single-point ribosomal mutants towards multi-aminoglycoside resistance
Source: PLoS Genet. 2025 Aug 25;21(8):e1011832. doi: 10.1371/journal.pgen.1011832 (PMC12416847; doi:10.1371/journal.pgen.1011832)

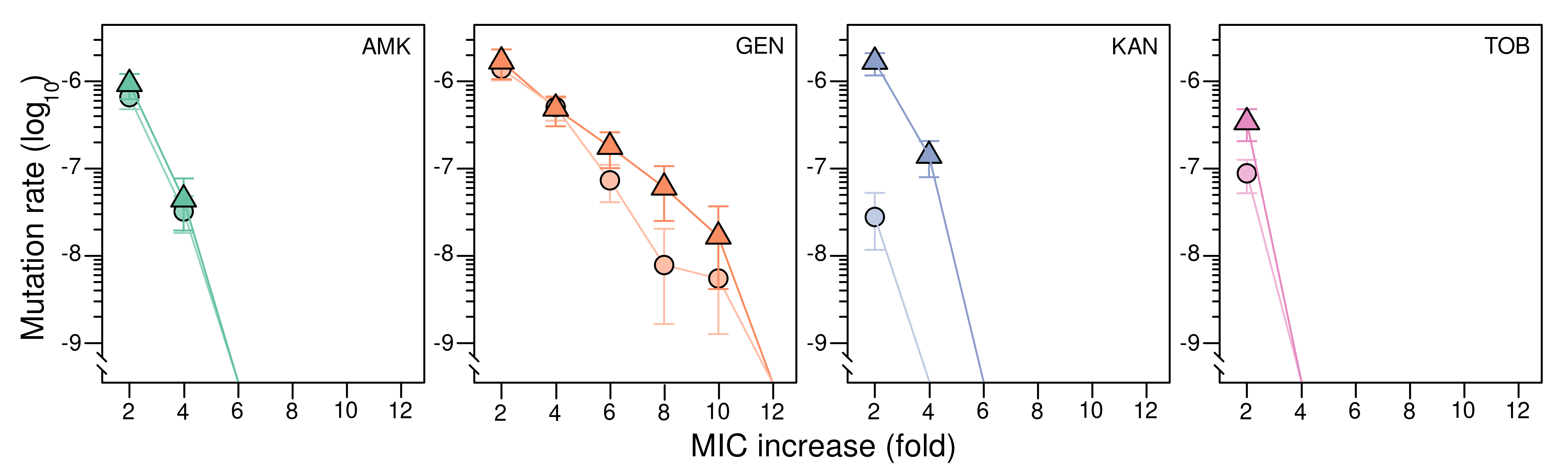

Supplement: S2 Fig — Mutation rate for 2-DOS aminoglycoside resistance in the K88E strain (triangles, darker colors) compared with the ancestral strain (circles, lighter colors) across a gradient of lethal concentrations of amikacin (AMK, green), gentamicin (GEN, orange), kanamycin (KAN, blue), and tobramycin (TOB, pink). Antibiotic susceptibilities are shown as fold increases relative to the Minimal Inhibitory Concentration (MIC) of the ancestral strain. Values represent the mutation rate ± 95% confidence interval from three replicates. (TIF) [file pgen.1011832.s002.tif]

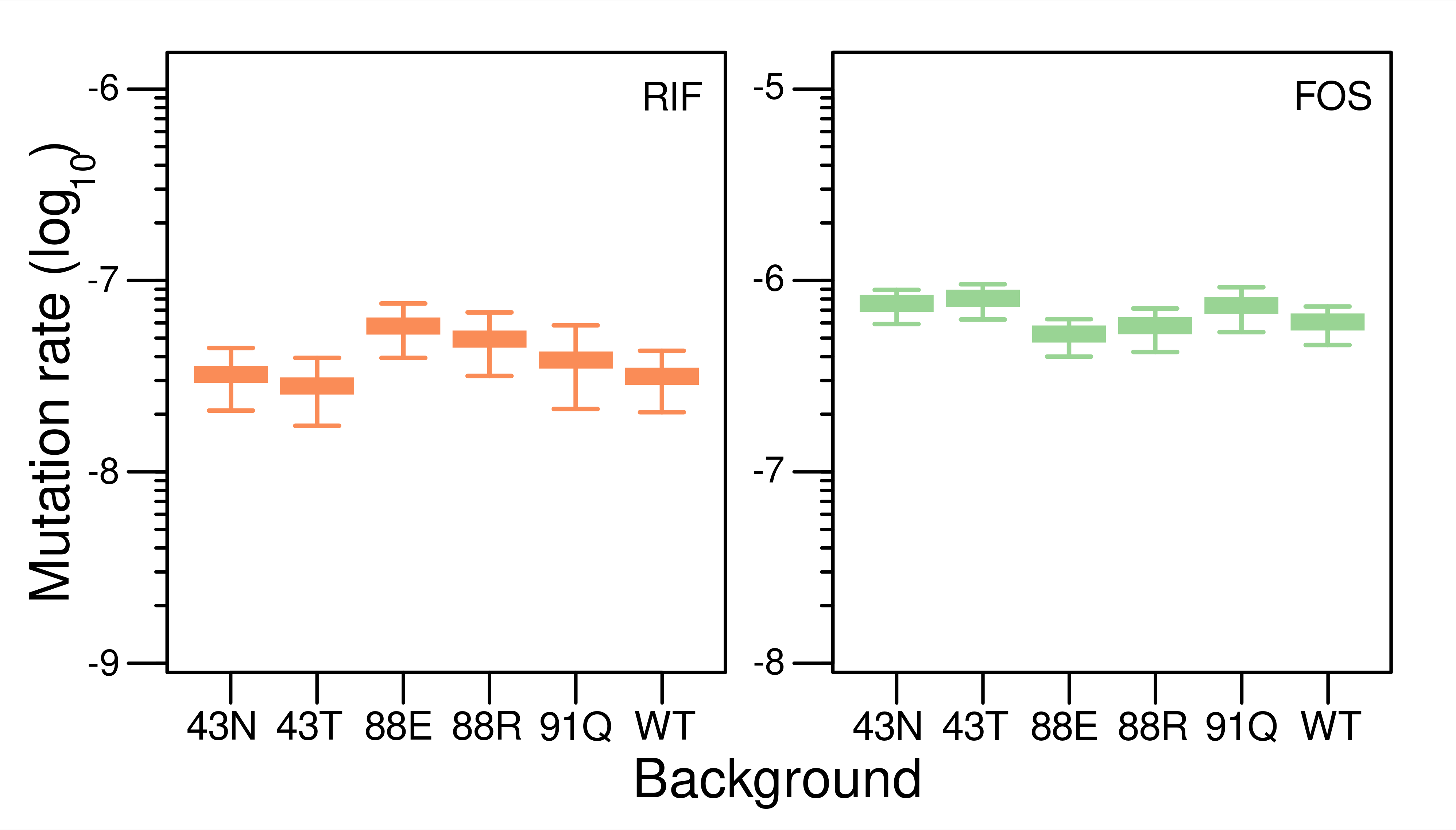

Supplement: S3 Fig — Rifampicin (RIF) and fosfomycin (FOS) resistance serve as proxies for genome-wide elevations in mutation rate, as RIF-resistant mutants typically arise from mutations in rpoB, located approximately 759 kbp from rpsL in the E. coli genome, while FOS-resistant mutants map to glpT and uhpT, approximately 1104 kbp and 404 kbp away, respectively. Values represent the mutation rate ± 95% confidence interval, estimated from five parallel replicate cultures. The small differences in mutation rates are not significant (pairwise likelihood ratio tests, Benjamini-Hochberg corrected). (TIF) [file pgen.1011832.s003.tif]

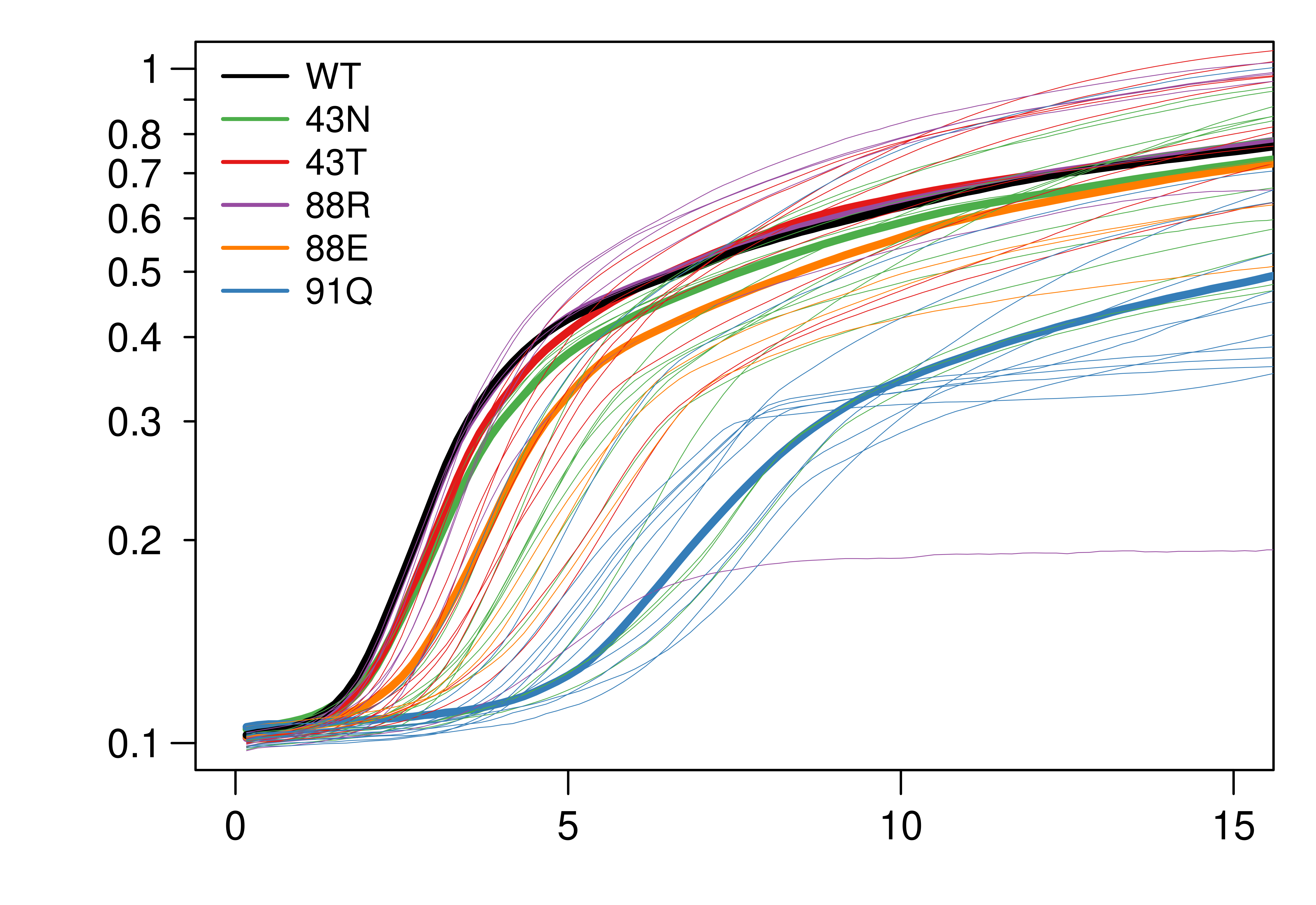

Supplement: S4 Fig — Each line represents the average optical density over time from three parallel assays per strain. Thick curves represent the ancestor (black) and the first-step mutants (43N, green; 43T, magenta; 88R, purple; 88E, orange; 91Q, blue). Thin curves correspond to the second-step mutants, following the color coding of their corresponding first-step mutant. Maximum growth rates were estimated using a custom script that calculates the maximum slope of the natural logarithm of optical densities versus time. The script analyzed the data using a sliding window of 1 h (six points, 10 minutes apart). Within each window, the script smoothed the data using a three-step moving average and then estimated the slope by fitting a linear regression model. (TIF) [file pgen.1011832.s004.tif]

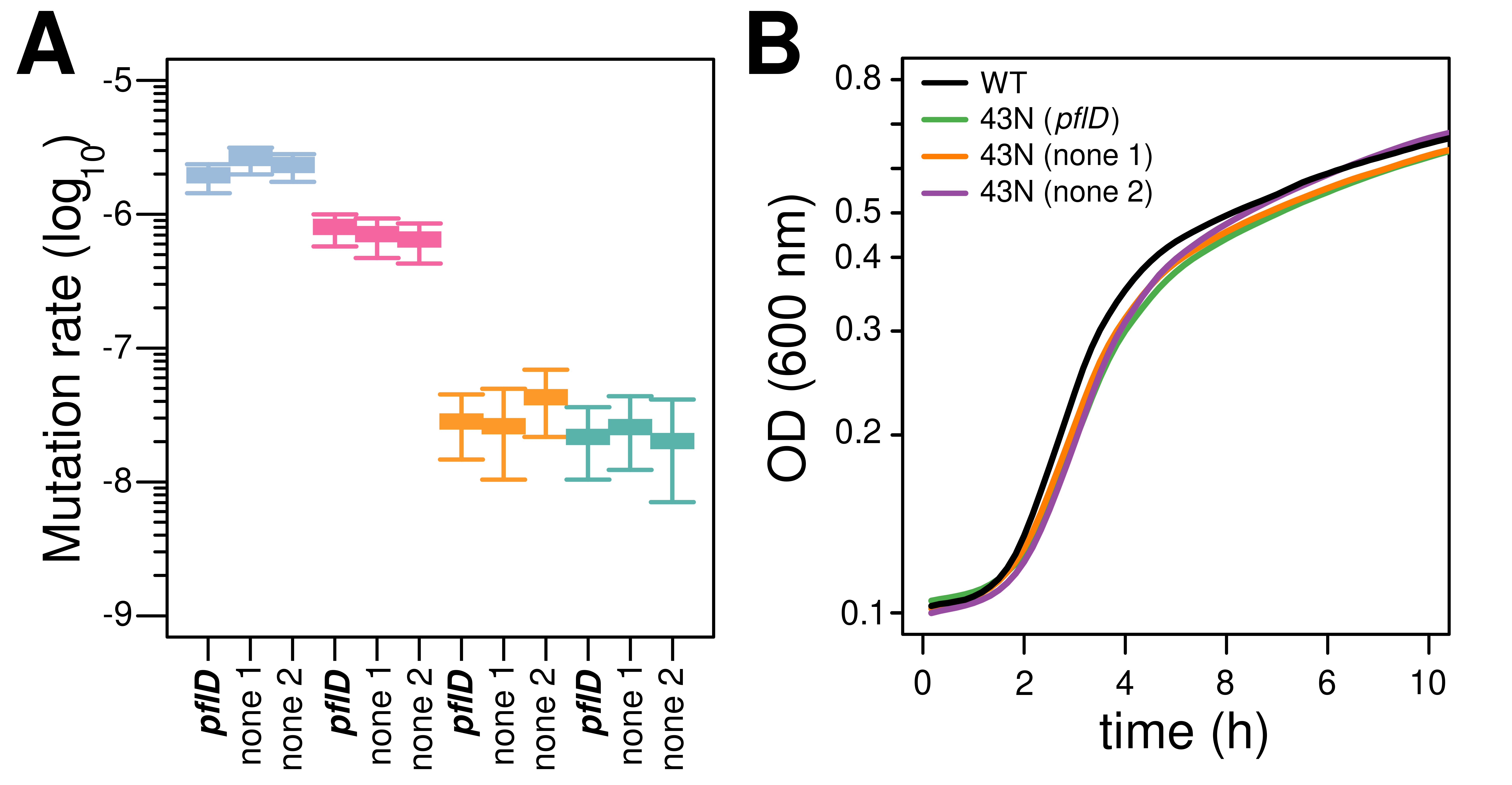

Supplement: S5 Fig — (A) Mutation rates for 2-DOS aminoglycoside resistance in the original K43N first-step mutant carrying the pflD mutation and two independently isolated clones confirmed to carry only the intended K43N rpsL mutation. Values represent the mutation rate ± 95% confidence interval, estimated from five parallel replicate cultures. Selection was performed at the wild-type’s Mutant Prevention Concentration (MPC), as in Fig 1. The small differences in mutation rates are not significant (pairwise likelihood ratio tests, Benjamini-Hochberg corrected). (B) Growth curves in the absence of antibiotics for the original K43N first-step mutant carrying the pflD mutation and the two clones confirmed to carry only the K43N mutation in rpsL. Details as in S4 Fig. (TIF) [file pgen.1011832.s005.tif]
