# Supplementary material for: Idiosyncratic evolvability among single-point ribosomal mutants towards multi-aminoglycoside resistance
Source: PLoS Genet. 2025 Aug 25;21(8):e1011832. doi: 10.1371/journal.pgen.1011832 (PMC12416847; doi:10.1371/journal.pgen.1011832)
Supplement: S1 Table — (DOCX) [file pgen.1011832.s006.docx]

**S1 Table. Effect on antibiotic susceptibility conferred by *pflD* R575S in the K43N background**

| **Antibiotic** | **MIC (µg/mL)** | | |
| --- | --- | --- | --- |
|  | K43N + *pflD*_R575S_ | K43N (clone 1) | K43N (clone 2) |
| **AMK** | 8 | 8 | 8 |
| **GEN** | 8 | 8 | 8 |
| **KAN** | 25 | 25 | 25 |
| **TOB** | 8 | 8 | 8 |
