# Supplementary material for: Idiosyncratic evolvability among single-point ribosomal mutants towards multi-aminoglycoside resistance
Source: PLoS Genet. 2025 Aug 25;21(8):e1011832. doi: 10.1371/journal.pgen.1011832 (PMC12416847; doi:10.1371/journal.pgen.1011832)
Supplement: S2 Table — (DOCX) [file pgen.1011832.s007.docx]

**S2 Table. Effects on antibiotic susceptibility conferred by fusA P610L across three backgrounds**

| **Antibiotic** | **MIC (µg/mL)** | | | | | |
| --- | --- | --- | --- | --- | --- | --- |
|  | WT | WT + *fusA*_P610L_ | K88E | K88E + *fusA*_P610L_ | K43N | K43N+ *fusA*_P610L_ |
| **AMK** | 8 | 8 | 8 | 32 | 16 | 200 |
| **GEN** | 8 | 32 | 8 | 8 | 8 | 200 |
| **KAN** | 12.5 | 50 | 25 | 50 | 25 | 400 |
| **TOB** | 4 | 4 | 8 | 4 | 8 | 64 |
