# Supplementary material for: Idiosyncratic evolvability among single-point ribosomal mutants towards multi-aminoglycoside resistance
Source: PLoS Genet. 2025 Aug 25;21(8):e1011832. doi: 10.1371/journal.pgen.1011832 (PMC12416847; doi:10.1371/journal.pgen.1011832)
Supplement: S3 Table — (DOCX) [file pgen.1011832.s008.docx]

**S3 Table. Primers used for CRISPR/Cas9-mediated gene editing**

| **Name** | **Sequence (5’ to 3’)** | **Use** |
| --- | --- | --- |
| fusA_500_Rev | CCGGCGCGTTATCGATCTGGTC | cloning and sequencing |
| fusA_500_Fw | GGGCGAACTGCACCTCGACATC | cloning and sequencing |
| fusA_SDM_Fw | GCTTGAGCCGATCATGAAAGTTGAAGTAGAAACTCCG | Site-directed mutagenesis |
| fusA_SDM_Rev | CGGAGTTTCTACTTCAACTTTCATGATCGGCTCAAGC | Site-directed mutagenesis |
| fusA_gDNA_Fw | AAACCGAAACCAGTTCTGCTTGAGCCGATCATGAG | pCRISPR-gDNA construction |
| fusA_gDNA_Rev | AAACTCATGATCGGCTCAAGCAGAACTGGTTTCG | pCRISPR-gDNA construction |
| pCRISPR_SC_Rev | GCCCAGTCATAGCCGAATAG | pCRISPR-gDNA screening |
| fusA_SC_P610_Fw | TGTAATCCCTGGCGAATACA | ARMS screening |
| fusA_SC_P610_wt | TCGGCTCAAGCAGAACTG | ARMS screening |
| fusA_SC_P610_mut | TCGGCTCAAGCAGAACTA | ARMS screening |
