# Supplementary material for: Idiosyncratic evolvability among single-point ribosomal mutants towards multi-aminoglycoside resistance
Source: PLoS Genet. 2025 Aug 25;21(8):e1011832. doi: 10.1371/journal.pgen.1011832 (PMC12416847; doi:10.1371/journal.pgen.1011832)
Supplement: S4 Table — (DOCX) [file pgen.1011832.s009.docx]

**S4 Table. Differences between the parental strain and the GenBank reference (U00096.3)**

| **position** | **mutation** | **annotation** | **gene** | **description** |
| --- | --- | --- | --- | --- |
| 547,694 | A→G | pseudogene (114/252 nt) | *ylbE→* | putative protein, C-ter fragment (pseudogene) |
| 547,835 | +G | pseudogene (4/1008 nt) | *ylbE→* | putative protein, C-ter fragment (pseudogene) |
| 1,976,527 | Δ776 bp | mobile element | *insB1–insA* | insertion sequence IS1 |
| 3,957,957 | C→T | intergenic (-21/+78) | *ppiC ←/← yifN* | rotamase C/conserved protein (pseudogene) |
| 4,294,404 | +GC | intergenic (+587/+55) | *gltP →/← yjcO* | glutamate/aspartate:proton symporter/hypothetical protein |
