# Supplementary material for: Idiosyncratic evolvability among single-point ribosomal mutants towards multi-aminoglycoside resistance
Source: PLoS Genet. 2025 Aug 25;21(8):e1011832. doi: 10.1371/journal.pgen.1011832 (PMC12416847; doi:10.1371/journal.pgen.1011832)
Supplement: Attachment — Submitted filename: PGENETICS-D-24-00553_Couce_response_letter.pdf [file pgen.1011832.s011.pdf]

Point-by-point response to reviewers for PGENETICS-D-24-00553

Dear Drs. Gordo and Crosson,

thank you and the reviewers for the careful attention to our paper. We have thoroughly addressed all the concerns raised, resulting in a much tighter manuscript. In addition to multiple in-text edits, we have redrawn Figure 1 with new mutation rate analyses, and added three supplementary figures and three supplementary tables with new experimental data to further support our findings. Notably, CRISPR-mediated gene editing of the most common second-step mutation in different first-step backgrounds revealed that differences in evolvability stem from strong positive (synergistic) epistasis. All remaining figures of the main text, as well as the former Figure S3 (now S4), were also redrawn as per reviewer's requests. Moreover, we now provide all numeric data underlying the figures in a spreadsheet, and have included the reference for sequencing files deposited at NCBI. Due to the workload involved in generating and analyzing these new data, as well as revising the manuscript, we would like to request that the second author be given shared consideration as a co-first author. Please find below a point-by-point response (in blue) to the reviewers' comments (in black). Text highlighted in yellow refers to changes in the manuscript. To facilitate tracking, edits in the revised manuscript file are highlighted in orange.

On behalf of the authors, sincerely,

Alejandro Couce

Reviewer #1:

The authors investigated the effect of first-step streptomycin resistance mutations in rpsL on the ability to evolve resistance to 2-DOS aminoglycosides. They observed that in most, but not all cases, first-step rpsL mutants have second-step mutations that are not accessible from the wild type at the selection concentration. The increase in resistance is lower for first-step mutants with higher initial resistance, consistent with a trend of declining adaptability. The authors also found substantial cross-resistance between mutants selected for 2-DOS aminoglycosides, while streptomycin resistance is largely retained. Growth in the absence of antibiotics is reduced in 4 out of 5 first-step mutants, a fitness cost that may be exacerbated or reduced in the second selection step. WGS revealed that second-step resistance mutations also target ribosomal proteins and components of the electron transport chain.

Thank you for this clear summary of our work and its main findings.

How existing genetic diversity affects the evolvability of resistance to antibiotics is an interesting and highly relevant question, important for the development of new strategies against resistance evolution. This work shows that readily accessible single-step streptomycin resistance mutations can facilitate adaptation to other aminoglycosides, which is potentially clinically relevant. The results are clearly presented and the experimental design adequately addresses the question posed. The conclusions are mostly well supported by the experimental evidence, with one major exception.

Thank you for highlighting the relevance of our study both from a fundamental and applied standpoint. We appreciate your positive assessment of our experimental design and clarity of our results. We addressed your concerns below.

Major comments:

1. Several first-step streptomycin mutants have been described as evolutionary dead ends that prevent the evolution of resistance to 2-DOS aminoglycosides. However, the experimental design only shows that several first-step mutants do not increase the evolvability over the wild type. Mutant

frequencies are concentration dependent in this assay and the choice of concentration therefore plays a key role. Here, the chosen mutant prevention concentration (MPC) of the wild-type, although justified to avoid mutants without the initial rpsL mutations, does not allow a more fine-grained comparison between the evolvability of the wild type and the first-step mutants in those cases where no mutants are observed at the MPC.

Thank you for this suggestion, which was also mentioned by Reviewer #3. To gain further insight into this issue, we have now measured the Mutant Prevention Concentration (MPC) for the most extreme case (K88E). The results, presented in the new Supplementary Figure S2, show that MPC values remain nearly identical across antibiotics, except for kanamycin, where a slight increase from 4 µg/mL to 6 µg/mL was observed. These findings suggest that the observed dead-ends stem from a failure to enhance evolvability beyond wild-type levels, rather than from a direct antagonistic interaction with secondary mutations. We have added a new paragraph in the manuscript reporting on these results (lines 210-215).

2. Similarly, the claim that the beneficial mutation rate is increased is doubtful, as most of the results can be explained by a reduced susceptibility to the respective aminoglycoside alone, allowing second-step mutations to overcome the resistance level with the same resistance increase that would be insufficient in the wild type.

Thank you for raising this important point. However, as per Reviewers #2 and #3 suggestion, we have now estimated true mutation rates instead of working just with mutant frequencies. Figure 1 has been updated with these new analyses, which show that the correlation between resistance level and evolvability is weaker than originally stated. We have now rewritten the final paragraph of the corresponding section (lines 231–234) to explicitly state that a reduced resistance hurdle only partially explains why less-susceptible genotypes are more evolvable than more-susceptible ones, suggesting an important role for idiosyncratic epistatic interactions between genetic backgrounds and secondary mutations. This change also removes the passage the reviewer found problematic (however, note that our original use of 'higher rates of beneficial mutations' was meant to refer to 'effective' mutation rates; we have now added this term in the two remaining occurrences—abstract and section (c) of the Results).

Minor comments:

1. Inconsistency in description of MIC change: Figure 1E and Figure 2 show different values for MIC fold increase. Presumably, Fig. 2 shows resistance values on a log2 scale. This needs to be clarified and would be easier to follow if it were consistent across different figures.

You are correct about the different scales. We have now updated Figure 2 to ensure consistency across figures.

2. Fix the inconsistency of the mutant order in the plot for 88R and 88E (compare e.g. Fig. 1 and 2).

Thank you for catching this issue. Labels in Figure 2 have now been corrected to match the alphabetical order used in Figure 1.

3. Lines 247-250: The reduction in streptomycin resistance in second-step mutants described for K88R is not visible in Figure 2, possibly because the color scale is saturated. The reduction for P91Q is drastic and visible, but only for KAN and TOB and not for all conditions.

Thank you for pointing this out, which was linked to your previous query. Indeed, only P91Q shows a reduction in three out of four second-step treatments—KAN, TOB, and partially in GEN. This passage has now been corrected.

4. The statistical description of correlations is somewhat unusual: the correlation is quantified by Pearson's R, while the p-value comes from a linear regression (e.g., in line 274 referring to Figure 3A). It would be more conventional to report the Pearson's correlation and the corresponding p-value and/or the slope of the regression and its significance.

Thank you for pointing out this possible source of confusion. R and other statistical software provide ready-to-use functions to run a t-test on the significance of the observed correlation (e.g., in R, *cor.test()*). This is mathematically equivalent to performing a t-test on the null hypothesis that the slope of a linear regression is zero. In practice, running *cor.test(x, y)* and fitting *lm(y ~ x)* in R yield the same p-value, as is illustrated and well-explained in this online example:

https://steverxd.github.io/Stat_tests/correlations.html#:~:text=lm%20. While we found it intuitive to report Pearson's R and state that the p-value comes from a linear regression, we recognize this may be an unnecessary complication. For clarity, we have removed references to linear regression from all instances of correlation tests throughout the text.

5. Line 260: (4/42, <1%) should read (4/42, <10%).

Thank you for catching this mistake. We have corrected it accordingly.

6. It is difficult to keep track of specific first-step mutants and individual replicates. Figure 1E and Figure 3 could be improved if the first-step mutants could be identified, e.g. by different symbols in the scatter plot. Also, in Table 1, the replicates cannot be traced to compare the fitness costs of the replicates in Fig. 4 with the observed mutations.

Thank you for highlighting this issue. We have revised Figures 1E and 3 as suggested, using different symbols to clearly identify first-step mutants. Additionally, to facilitate comparison between Table 1 and Figure 4, we have added fitness values as a new column in Table 1.

7. The determination of mutant frequencies is not described in the Methods section.

As stated above, and following the suggestions of Reviewers #2 and #3, we have now estimated true mutation rates instead of relying solely on mutant frequencies. As a result, we have updated Figure 1 and Figure S2, and we have added a corresponding description to the Methods section.

8. Since the fitness cost of resistance increase was measured, is there a correlation between null fitness and MIC of replicates?

We did look at the correlation between relative fitness and MIC change compared to the wild type but found it to be both weak and non-significant (Pearson's R = -0.203, P = 0.197). Given this, we opted not to include it in the manuscript. In any case, the full dataset is now available in the supplementary spreadsheet for readers to explore if desired.

<u>Reviewer #2 (attached):</u>

Sánchez-Maroto et al investigate the evolvability of E. coli MG1655 towards aminoglycoside resistance in a multi-step selection setup. The results reveal epistatic interactions (on fitness and resistance level) between resistance mutations. Additionally, they identified several first-step mutations that pose an evolutionary dead-end towards further resistance selection under the used experimental conditions. The authors discuss their work in a broader context of declining adaptability and diminishing returns of beneficial mutations, and how rpsL mutations can act as stepping-stones for resistance towards other antibiotics than streptomycin. The identification of epistatic interactions, and the 'priming' of streptomycin resistant mutants towards resistance to other antibiotics is novel and exciting, however there are issues with major claims of the study as well as the experimental execution:

Thank you for this clear summary of our work and for highlighting the novelty and excitement of our findings. We address your concerns below.

Major

The authors lay out the concept of a declining adaptability and diminishing returns and aim to investigate if antibiotic resistance is underlying these principles. I don't believe the experimental setup is adequate to answer this question: First, the selective pressures in the study are not consistent, as first step and second step mutations are not selected under the same selective pressure, but rather by (i) streptomycin followed by (ii) different aminoglycosides challenge. Second, not only the selective pressure applied, but also the read-out for 'fitness' changes, from streptomycin resistance to resistance (or even mutation frequency) to different antibiotics. Given this, I find an interpretation of the presented results in regards to declining adaptability challenging, and somewhat misleading. Lastly, it is well known that antibiotic resistance genes/mutations often show positive epistatic interactions, even for streptomycin resistance under a constant selective pressure (PMID: 29686259). Thus, the authors premises and motivation such as to "clarify wether declining adaptability patterns … are general phenomena in antibiotic resistance" (Lines 130+131) are confusing and their novelty limited.

Thank you for your feedback, we will address your three points in order. First, an standard way to study declining adaptability patterns and evolvability suppressors is to generate mutant strains, characterize their genotypic and phenotypic features (e.g., mutations, initial susceptibility), and subsequently measure their capacity to overcome antibiotic challenges. Importantly, the method used to generate the initial mutant collection does not have to match the subsequent antibiotic treatment. For instance, Lukačišinová et al. (2020, Nat Commun) and Horinouchi et al. (2020, Sci Rep) created such collections by engineering full-gene knockouts, while Card et al. (2019, PLoS Biol) obtained their test strains through long-term evolution in drug-free conditions. Similarly, we created our panel of mutants through initial exposure to streptomycin—which is arguably a more realistic way to generate starting mutants than engineering genome-wide knockouts. We have now made this explicit by adding a sentence in section (c) of the Results.

Second, a standard approach for testing declining adaptability is plotting initial versus final susceptibility levels to the same antibiotic (e.g., Fig 2b in Lukačišinová et al., 2020, Nat Commun; Fig 2a in Horinouchi et al., 2020, Nat Commun; Fig. 3 in Card et al. 2019, PLoS Biol). This is exactly what we report in Figure 3. From your comment, it seems there may have been a misunderstanding about what this figure actually compares. To clarify, we have revised Figure 3's caption to explicitly state that comparisons are made between initial and final susceptibility for each 2-DOS aminoglycoside.

Finally, as explained in the paragraphs preceding the sentence you alluded to, a key goal in this field is to identify genetic features that predict a reduced capacity for antibiotic resistance evolution (refs. 48–53). Two phenomena of growing interest are declining adaptability patterns and evolvability suppressors, often reported separately. Our work shows both are common in a novel model system—resistance to 2-DOS aminoglycosides. Moreover, this system offers key advantages, including initial strains that differ by single-point mutations—the smallest possible genetic change—and the ability to test evolvability across multiple relevant phenotypic axes. We do not see how the report of both positive and negative epistasis being common among all possible combinations of five mutations linked to streptomycin resistance (PMID: 29686259) would undermine the novelty of our premises or motivation Actually, note that negative epistasis is what is often invoked to explain declining adaptability patterns, so if anything, the possibility of positive epistais makes our study even more

pertinent. Nevertheless, we agreed to revise the sentence in question to clarify that our goal is to help elucidate how common declining adaptability patterns and evolvability suppressors are, rather than to establish them as general phenomena.

The study falls short on any attempt to a mechanistic explanation. It would be interesting to see constructed double mutants of supposably non-viable resistance pathways (a dead-end rpsL mutation with a second step resistance mutation), to determine if resistance can not evolve due to synthetic lethality even in absence of drugs or because no further resistance increase is provided. The authors hint to a potential explanation linked to "increased avoidance of macrophages immune proteins", "reduced rates of protein oxidative damage" or different translation rates (lines 453-454), but do not perform any experiments towards this end.

Thank you for this valuable suggestion. We have now used CRISPR-mediated gene editing to introduce the most common second-step, single-point mutation (*fusA* P610L) into both the wild-type and the less evolvable first-step background (*rpsL* K88E). We then compared its effects in these dead-end backgrounds to its effect in the most evolvable background (*rpsL* K43N). Our results indicate that synthetic lethality is not the limiting factor; rather, strong epistasis underlies the differences in evolvability. These new findings are now presented in Table S1 and discussed in a newly added section (d) in the Results. We have also included a brief mention of these new results in the Discussion, as well as a new section (g) in Methods describing the CRISPR/Cas9-mediated gene editing procedures. Lastly, we do not suggest increased avoidance of macrophages or reduced protein oxidative damage as potential explanations for our results. As explicitly stated in the sentence before this passage, these references serve to illustrate that ours is not the first report of idiosyncratic behavior of mutations at this site.

The authors discuss their findings in the context of clinical implications, warning that rpsL mutations could prime pathogens for resistance towards other aminoglycosides. To this end, a literature review, or sequence analysis of clinical isolates, would be helpful to understand at what frequency, if at all, the mentioned pathogens (P. aeruginose and A. baumanii (lines 492+493) carry mutations in rpsL, suggesting a contribution to GEN or TOB resstance in the clinics. Is resistance to TOB, KAN, GEN,

AMK typically acquired via chromosomal mutations rather than aminoglycoside modifying enzymes? Are the reached resistance levels clinically relevant?

We agree that these questions are highly interesting; however, addressing them properly would require a full-fledged study beyond the scope of this paper. Consequently, we have removed the explicit reference to GEN and TOB as the treatment of choice for *P. aeruginosa* and *A. baumannii*.

As acknowledged in line 183+184 the five different streptomycin-resistance mutations display a range of fitness effects in absence of the drug. However, the selection was performed without OD (or CFU) normalization. How are mutation frequencies calculated, without determining the starting amount of cells? A different population size will consequently result in different amounts of mutants even without allel specific differences in 'evolvability'. At a minimum, the authors should repeat some of the selection experiments with OD normalization to show that the observed effects are not affected by different overnight yields.

Thank you for raising this concern about how unequal final population counts could affect our estimation of mutant frequency as a proxy for mutation rates. However, recent work demonstrates that under typical experimental conditions, variations in final population size have a small to negligible impact on mutation rate estimates (Zheng 2016, *J. Theor. Biol.*). That said, in response to your comment and a related one by Reviewer#3, we have now used rSalvador, a state-of-the-art software, to directly estimate true mutation rates rather than settling solely on reporting mutant frequencies. Of note, rSalvador includes algorithms that corrects for unequal final population counts when calculating mutation rates. As a result, we have updated Figure 1 and Figure S2, and we have added a corresponding description to the Methods section.

Materials+Methods section on how mutation frequencies were determined are missing. Assuming the data simply reflects the number of mutants per population, it is not suitable to determine the effect of rpsL mutations on genome wide spontaneous mutation rates (unlike fluctuation or mutant accumulation tests).

As explained in response to the preceding point, we now report true mutation rates. Figure 1, Figure S2 and the Methods section have been updated accordingly.

Line 520-525: The authors state that the rpsL K43N mutant carried an off-target mutation in pflD. To verify the phenotype, two other K43N mutants that did not carry a pflD mutation were tested and behaved similarly to the pflD/rpsL double mutant. Given that mutations in pflD gene have previously been linked to rpsL mutations causing streptomycin resistance (https://doi.org/10.1021/acsinfecdis.3c00156), the authors should (i) adress the presence of the off target mutation in the main result section, rather than in mat/met, (ii) whole genome sequence the two K43N clones used for validation to ensure absence of any off-target mutation (especially considering the high-amounts of off-target mutations observed in the second step mutants) and (iii) report the data (susceptibility profile, fitness, mutation frequency) of the two additional clones that is referred to in lines 523+524.

These two independent clones have now been sequenced, confirming the absence of secondary mutations. Their sequences have been deposited in the NCBI SRA. Additionally, we have included a new Table S3 to show the similarity in susceptibility profiles and a new Figure S5, which contains two panels: one comparing mutation rate profiles across antibiotics and the other growth curves – in both cases clearly supporting the similar behavior of all three clones. After careful consideration, we still believe that these technical details are best placed in the original Methods section to maintain the readability of the main text.

Figure S3: According to Methods, growth rates were determined with a starting OD of 0.01, however, starting OD's are 0.1 in the figure, suggesting that a background subtraction was not performed. It is also confusing why only the ancestor growth curve starts at time 0, while all others are somewhat delayed. Additionally, the growth curves appear to be linear, but falsely plotted on a logarithmic scale. If correct as plotted, the strains (even the wt) have an unusually long doubling time (>1h?), and a large OD increase during stationary phase. I suspect that during image editing a (fundamental) mistake slipped in, please double check that the presented data is correct and fits the displayed scale.

Thank you for your careful attention to this figure. You are correct that we did not subtract the blank from the optical density (OD) measurements. We find it more intuitive to display the y-axis on a logarithmic scale (as the maximal growth rate during exponential growth is extracted from the steepest

part of the log-transformed curve). Subtracting the blank leads to stochastic negative values at the beginning of the growth curves, creating a misleading appearance. We also appreciate your observation regarding the apparent delay in mutant growth curves compared to the ancestor. To clarify, all curves start at 0.167 hours (10 min). However, there was a minor issue where the smoothing moving window algorithm was applied unequally among the datasets, which has now been corrected. Regarding your concern about the shape of the curves, we confirm that the growth curves are correctly displayed. Doubling times of around 1 hour are not uncommon for *E. coli* lab strains grown in microplates, as oxygen limitation (due to restricted aeration and surface tension effects) slows down growth compared to well-aerated flasks. Similar doubling times can be seen in other studies (e.g., Fig. 1A in Krishnamurthi 2021, *PLoS One* 16; Fig. 2A in Kotecka 2021, *Int. J. Mol. Sci.* 22; Fig. 1A in Couce 2012, *Antimicrob. Agents Chemother.* 56). The observed OD increase during stationary phase is also not unusual and is likely due to significant light scattering effects in microplate measurements at high cell densities (see the figures referenced above; Krishnamurthi et al. 2021 includes an explicit discussion of multiple scattering). Besides redrawing Figure S3, we now provide the underlying data in the supplementary dataset.

Figure 3 needs clarification. The colour scheme shows what the mutants were selected on, but it is not fully clear what the MIC values refer to. Assuming that ‘MIC first’ and ‘MIC second / MIC increase’ refer to streptomycin and TOB, AMK, GEN, KAN, respectively, it should also be clarified what the MIC increase refers to. Is it, for example, increase in TOB MIC of the second step mutant towards the first step mutant, or towards the original ancestral strain?

As already said in the response to your first comment, we have revised Figure 3’s caption to explicitly state that comparisons are made between initial and final susceptibility for each 2-DOS aminoglycoside.

Minor

Figure 1D: AMK doesn’t match Figure 1C – I assume 88E and 88R are mixed up.

Thank you for catching this mistake. We have now corrected these panels.

Line 511: Should be 100mg/L instead mg/ml.

Thanks. We have corrected it accordingly.

Figure 1C) Indicate the concentrations (or rather fold X MIC) that the second step mutants were selected on.

Explicit mention of the fold X MIC used has now been added to the Figure's caption.

Line 163: Authors state that the susceptible strains exhibit lower mutation rates, but later on state that the difference in 'evolvability' is not due to differences in mutation rates. Please clarify, or correct if wrong wording.

We use the phrase 'higher rates of beneficial mutations' to implicitly refer to 'effective' mutation rates. Figure S2 confirms that there is no genome-wide increase in general mutation rates. Thus, the observed increase in 2-DOS-aminoglycoside resistance mutations in certain mutants must stem from their greater accessibility to secondary adaptive mutations. In other words, this effect is driven by epistasis rather than an elevated replication error rate, stress-induced mutagenesis, or similar mechanisms. To clarify this point, we have now explicitly added the term 'effective' throughout the text (abstract and section (c) of the Results).

Line 557: Should be -80° freezer instead of 80° freezer.

Thanks. We have corrected it accordingly.

Figure 2) As stated in line 232, the experiment to determine cross-resistance and collateral sensitivity were performed in triplicate. Please clarify how the results were handled in cases were replicates disagreed

Results are reported as the median of three replicates. This is now explicitly stated in the corresponding Methods section (f).

Figure 2) The maximum fold increase is indicated as 10x. Is this really the actual increase in MIC or just the limit of detection? I was under the impression most rpsL mutations confer a much higher MIC than 10x wild type, almost limited by streptomycin solubility. Please clarify and add a table with all MIC values for each genotype.

Thank you for spotting this omission. Figure 2 depicts resistance values on a log_2 scale, but this was not properly indicated in the legend. We have now updated the Figure. As you noted, the actual limit is >256-fold the MIC, which is indeed close to the solubility limit of streptomycin.

Figure 4) The number of replicates should be stated in the figure legend, and standard deviations should be shown. It would be good to add information, which of the strains carried off-target mutations (which likely act as compensatory mutations) in the figure.

We have updated the figure caption to include: "Values represent the mean of three replicates. Standard deviations are small and omitted for clarity but are available in the supplementary dataset. Strains subjected to whole-genome sequencing are indicated with a thicker line".

As fairly stated by the authors, the frequent occurrence of off-target mutations in the selected strains is puzzling (lines 376-390). I agree with the arguments, that a stochastic effect is very unlikely, and that the off-target mutations must have appeared after selection as compensate potential fitness deficits. Since strains were grown in liquid to be cryo-preserved as well as for whole genome sequencing, selection for increased growth was not only present during the initial selection, thus I believe the authors comment that "the second mutation became predominant within the colony as it grows on the selective plate" (lines 388+389) is too speculative.

Thank you for this observation. Please note that we do not argue that these second-site mutations are merely compensatory (i.e., alleviating growth impairment in the absence of antibiotics). Rather, we suggest they are further adaptive mutations that enhance resistance and thus promote growth. This is supported by their occurrence in genes related to the ribosome or the electron transport chain. While some compensatory mutations may arise, particularly in the most impaired backgrounds (e.g., *xdhA* in

P91Q), selective pressures favoring increased resistance are likely more significant. Nonetheless, we have added a brief mention of this possibility in the Results section (e) .

Reviewer #3:

The manuscript entitled "Idiosyncratic evolvability among single-point ribosomal mutants towards multi-aminoglycoside resistance", by Sánchez-Maroto et al., aims to determine whether the presence of streptomycin resistance mutations can favor or hamper appearance of additional mutations that confer resistance to other aminoglycosides. The main conclusions of the authors are that (i) most streptomycin resistance mutations studied facilitated evolution of resistance to other aminoglycosides, (ii) in a few cases, this did not happen, (iii) more fit genotypes showed higher rate of acquisition of beneficial mutation, but smaller effect sizes than less fit genotypes, (iv) second-step resistance mutations mostly confer cross-resistance across aminoglycosides, and (v) in some cases, second-step resistance mutations can increase or decrease the fitness cost caused by first-step resistance mutations.

Thanks for your clear summary of our study and its key findings.

The manuscript clear, well written and easy to follow, being therefore accessible to the broad readership of PLOS Genetics. The observations described in the manuscript are timely and relevant, as the observed increased evolvability of antibiotic resistant strains with respect to susceptible clones represents an additional challenge in the already complex antibiotic resistance crisis. However, I have a number of concerns that, if addressed, would ratify that the conclusions reached by the authors are properly supported by the data provided. These concerns are discussed below, divided as major and minor, and organized by relevance. Line numbers refer to those in the pdf version of the manuscript.

Thank you for recognizing the relevance of our study. We also appreciate your positive assessment of the clarity of our results. Below, we address your concerns in detail.

MAJOR CONCERNS

1. The fact that most first-step streptomycin resistant mutants can evolve resistance to aminoglycosides at concentrations that their susceptible ancestor can not is the core observation of the study. A possible explanation for that observation is that overall mutation rate of streptomycin resistant mutants is higher than that of the susceptible ancestor. In order to rule out this possibility, authors estimated mutation

rates by calculating the frequency of appearance of rifampicin resistant mutants in all genotypes (Figure S2). I see two problems with this experiment:

i) Strong epistatic interactions between rifampicin and streptomycin resistance mutations have been repeatedly observed, which causes some streptomycin and rifampicin resistant mutants to show large fitness costs (PMID: 1094452; PMID: 322146; PMID: 19629166; PMID: 23206139; PMID: 26130082; PMID: 28419091; PMID: 33830249). Thus, in certain streptomycin resistant backgrounds, spontaneous appearance of rifampicin resistant mutants in cultures can show a large fitness disadvantage, which influences their frequency.

ii) Based on the legend of the axis in Figure S2, I assume (this experiment is not described in Materials and Methods) the experiment measured frequency of appearance of resistant mutants instead of mutation rates (in which the aforementioned frequency is normalized to the number of generations in each genotype). This is problematic, considering that growth rates of streptomycin resistant genotypes largely differ from that of the ancestral susceptible strain (Figure S3 and Figure 4).

Because of the two reasons above, and considering the importance of measuring mutation rates for the key discoveries described in the manuscript, I think mutation rates should be properly estimated by selecting for mutants that show no epistatic interactions with streptomycin resistance mutations (e. g. nitrofurantoin resistance mutations), and normalizing their frequencies of appearance to the number of generations that each genotype underwent during the experiment. Proper estimation of mutation rates of genotypes carrying the K43T, K43N and K88E substitutions is particularly important, as these three genotypes have been demonstrated to show increased SOS induction (PMID: 33830249), which typically increases mutation rate.

Thank you for these valuable suggestions. To answer your first point, we were initially satisfied with rifampicin because mutant frequencies did not differ from the wild-type: it seemed unlikely that strong epistatic interactions between streptomycin and rifampicin resistance mutations would precisely counterbalance any potential increase in overall mutagenesis. However, following your suggestion, we have now included a second mutagenesis marker (fosfomycin resistance) to further verify whether

overall mutation rates remain unchanged. The results confirm our initial conclusion and are now presented as an additional panel in Figure S2.

Regarding your second point, we agree that estimating true mutation rates provides a more robust assessment than relying solely on mutant frequencies. To address this, we have now used rSalvador, a state-of-the-art software that directly estimates mutation rates while accounting for differences in the number of generations each genotype undergoes. As a result, we have updated Figure 1 and Figure S2, and added a corresponding description to the Methods section.

2. I find surprising that the streptomycin resistant alleles selected in the firs-step selection were not transferred to a clean background by P1 transduction, which is technically trivial and would dramatically decrease the chances of these genotypes to carry additional mutations (such as compensatory ones) together with the ones conferring resistance to streptomyicin. This is particularly relevant, considering that almost half of the 18 isolates that were subject to whole genome sequencing showed two mutations apart from the one conferring resistance to streptomycin. Indeed, sequencing of the strain carrying the K43N substitution in RpsL showed an additional mutation (discussed in section b of materials and methods, lines 518-525), demonstrating that mutations other than those conferring resistance to streptomycin were selected during the process of obtaining the first-step mutants. It is not clear whether or not all first-step streptomycin resistant genotypes were subject to whole genome sequencing and, if yes, what were the results of such analysis (number of genotypes showing additional mutations, number and nature of those mutations, etc.). One could argue whole genome sequencing of fist-step streptomycin resistant mutants is required in order to perform a proper comparison with those of second-step mutants; I was surprised not to find this data in the manuscript. In any case, since the conclusions of this work are based on the assumption that all observed differences between the susceptible ancestral and first-step mutants (in evolvability, fitness, etc.) are due exclusively to the presence of the streptomycin resistant alleles, it is paramount to determine if any other streptomycin resistant first-step mutant carries additional mutations, how many, which ones, etc.

Thank you for raising this important issue. We address your two main points in order. First, we chose not to use P1 transduction (or CRISPR-based mutagenesis, for that matter) precisely to avoid introducing further secondary mutations. For one thing, P1vir infection can be mutagenic as it activates

the SOS response (Cech 2021, *Int. J. Mol. Sci.* 7). Additionally, since some first-step mutants exhibit substantial fitness costs, subjecting them to multiple rounds of growth, stress, and selection could increase the likelihood of acquiring compensatory secondary mutations. Therefore, we opted for a more direct approach—whole-genome sequencing (discussed below).

To your second point, we performed whole-genome sequencing on 18 strains derived from these first-step mutants. If any secondary mutations had arisen during the initial selection, they would have been consistently inherited by all derivatives of the corresponding background. This is exactly what allowed us to identify the *pflD* mutation as a presumed passenger—it appeared in all derivatives of the K43N background (see new data in Table S3 and Figure S5 supporting this interpretation). Aside from this exception, no other secondary mutations were consistently present across derivatives of any other background. The absence of such mutations strongly suggests that no additional secondary mutations were initially present—unless one assumes their simultaneous reversion in all derivatives, which seems highly improbable. We have also explicitly clarified this reasoning in the revised Methods section (b).

3. Connected with point 2 above, selection of first-step mutants was performed following two overnight growths of susceptible bacteria. Indeed, according to section b of materials and methods (lines 508-511), seems like a unique bacterial culture was grown overnight and then multiple dilutions of the very same culture were grown overnight again, prior to plating onto LB agar supplemented with streptomycin, to select for mutants. I see two main issues connected with this experimental procedure:

i) first, if all experimental replicates come from a single overnight culture, streptomycin resistant clones appearing in that single culture will propagate to the replicates, biasing the results towards increased relative abundance of the specific streptomycin-resistant allele selected in the first growth. The same would occur with any other mutation (either compensatory or highjacker) that may arise in that initial culture. Indeed, the second growth might select for compensatory mutations to streptomycin-resistant mutants that may have appeared in the first growth.

ii) second, he fact that bacterial cells are grown during two consecutive overnight periods dramatically increases the chances of appearance and selection of mutations.

This approach is even more problematic in the case of the second-step selection, which was done in a similar manner. In that scenario, streptomycin resistant mutants were grown in two consecutive overnight periods prior to selection on plates containing different aminoglycosides. The two consecutive growth periods make very likely accumulation of mutations that compensate for the fitness cost caused by streptomycin resistant allels, especially for the genotypes with lower fitness.

Thank you for your careful attention to our protocol. First, note that we perform a 1:10^{-4} dilution when seeding independent replicates. This is standard practice in fluctuation experiments and is specifically designed to eliminate any mutants that may have arisen in the initial overnight culture. Given that streptomycin-resistant mutants typically appear at a frequency of ~10^{-9}, an overnight culture would, at most, contain a few dozen mutants. With our dilution, the probability of sampling preexisting mutants is extremely low (~0.001), making it highly unlikely that they were carried over to the second overnight. Regarding the potential selection of compensatory mutations in this second overnight, we do not expect this scenario for the same reason—the probability of seeding a preexisting mutant is exceedingly low. Moreover, as outlined in our response to your previous point, whole-genome sequencing strongly suggests that no secondary mutations were initially present. To further clarify this, we have now explicitly stated the importance of the dilution step in the revised Methods section (b).

As for the possibility of compensatory mutations arising during second-step selection, whole-genome sequencing data suggests that secondary mutations are mostly contributing ro resistance rather than being compensatory. This is supported by their occurrence in genes associated with the ribosome or the electron transport chain (and makes sense: selective pressures favoring increased resistance are likely way stronger than those favoring compensation). Nonetheless, we now explicitly acknowledge in the Results section (e) that some second-step mutations may have a compensatory role, particularly in the most impaired background (e.g., *xdhA* in P91Q).

4. Certain streptomycin resistant mutants fail to evolve resistance to certain antibiotics at the concentration used in this study (Figure 1C). The authors interpret this as evolutionary dead-ends. I do not think this interpretation is supported by the data presented in the manuscript, as this selection was done at concentrations were the ancestral susceptible strain is not able to develop resistance either. According to the author's interpretation, this would imply that wild-type E. coli represents a

evolutionary dead end for aminoglycoside resistance mutations, which I do not think is a correct notion. In order to prove that these streptomycin resistant mutants unable to develop resistance to aminoglycosides are truly evolutionary dead-ends, the Mutant Prevention Concentration (MPC) should be determined for these genotypes (as it shown in Figure 1B). Only if MPC of these particular streptomycin resistant alleles is significantly lower than that of their susceptible ancestor, the intepretation of these genotypes as evolutionary dead ends would be substantiated.

Thank you for raising this interesting semantic point. In the context of our study, we do consider the wild-type a dead-end under the specific conditions used for second-step selection (by design, the wild-type's MPC). The term "evolutionary dead-end" is intended as a counterpart to the "stepping-stone" concept, both of which serve as metaphors to make our findings more intuitive and easy to recall for readers. If certain backgrounds provide the only viable path for further evolution (the "stepping stones", then, logically, the others must be "dead-ends".

In any case, we find your suggestion of estimating the MPC for some of the "dead-end" mutants very interesting (it was also suggested by Reviewer #1). We have now done so for the most extreme case (*rpsL* K88E). The results, presented in the new Supplementary Figure S2, show that MPC values remain nearly identical across antibiotics, except for kanamycin, where a slight increase from 4 µg/mL to 6 µg/mL was observed. These findings suggest that the dead-end nature stems from a failure to enhance evolvability beyond wild-type levels rather than from a direct antagonistic interaction with secondary mutations. We have added a new paragraph in the manuscript reporting these results (lines 210–215).

MINOR CONCERNS

A. Fitness of the all genotypes used for virulence experiments were estimated by obtaining growth curves based on optical density at 600nm (Figure S3). Albeit fast and simple, this method is prone to artifacts, since optical density only correlates with cell density at low values (PMID: 322614) and is affected by bacterial cell size and shape (discussed, for instance, in PMID: 36228033), both of which can be altered in mutants (PMID: 28886685). In my opinion, relative fitness measurements would be

more reliable if a more accurate method, such as CFUs measurement or competitive fitness assays, would be used.

We agree that microplate-based growth rate estimates have limitations such as the potential artifacts you mention. However, we believe these issues are unlikely to significantly affect our experimental settings. For instance, the maximum growth rate in our curves typically occurs early, within an OD range of 0.15–0.25—well within the "safe zone" shown in the reference you cite (PMID: 322614). Additionally, while differences in cell shape could influence optical density measurements, we have no strong reason to believe this applies to the single-point *rpsL* mutations studied here (in fact, Figure S2 in Balbontín 2021, *Mol. Biol. Evol.* 38 shows that the *rpsL* K43N mutant has a size and shape similar to the wild-type).

That said, we acknowledge that growth rate estimates are not a perfect proxy for fitness. Competitive fitness measurements integrate all growth phases, meaning factors such as lag time or stationary-phase survival can also play a role. However, when exponential growth rate is the predominant determinant of fitness—as is often the case in nutrient-rich laboratory media—higher maximum growth rates strongly predict relative fitness in direct competition assays (as explicitly demonstrated in Venkataram 2020, *PNAS* 117). We have now added a passage briefly discussing this in the Methods (e) section.

B. I am surprised the allele causing the substitution K43R did not appear among the streptomycin resistant genotypes, as it is a commonly selected allele and, importantly, it does not cause any defect in fitness, which intuitively would cause it to be fairly represented in any cultura it may appear, compared to other alleles causing large fitness costs. What's the author's interpretation of this result?

We are aware of the K43R substitution confering streptomycin resistance, but mostly in mycobacteria (PMID: 20951172, PMID: 19519851, PMID: 8913445). In *E. coli*, the classic study by Timms (1992, *Mol. Gen. Genet.* 232) found no evidence of K43R arising either spontaneously or under UV mutagenesis, reporting instead K43E, K43N, and K43T. Likewise, in our previous study on hypermutators with different mutation spectra (Couce 2013, *PLoS Genet. 9*), we observed K43N and K43T but not K43R. We may speculate that this absence may be due to different synonymous codons present at this position in different strains, which could influence the accesibility of a K43R mutation.

Moreover, epistatic interactions with other polymorphisms in *rpsL* or other ribosomal genes may also constrain its appearance in the particular strain of *E. coli* we used.

C. I am similarly surprised the allele causing the substitution P91Q was not classified as streptomycin dependent, considering that has been previously described as such (PMID: 8412684). How do the authors explain this disagreement?

In our previous study with hypermutators (Couce 2013, *PLoS Genet.* 9), we observed that the P91Q substitution was not streptomycin-dependent. However, Timms (1992, *Mol. Gen. Genet.* 232) did not even find it. As with the absence of K43R, we can only speculate that these discrepancies may stem from epistatic interactions with inevitable polymorphisms in *rpsL* or other ribosomal genes present across strains.

TEXT CORRECTIONS AND SUGGESTIONS

Lines 221-222. the text says: "Our analysis revealed a moderate positive correlation between initial susceptibility and mutant frequencies". The analysis shown in panel E of Figure 1 compares mutant frequencies with change in MIC. I suggest to correct the sentence above.

Panel E of Figure 1 indeed compares initial susceptibility with (now) mutation rates. The label “MIC increase (fold)” in the x-axis refers to the initial susceptibility of each single-step background.

Line 223. I believe the authors mean Figure 1E (there is no panel H).

Thanks for catching this typo. We have corrected it accordingly.

Line 225. the text reads "higher rates of beneficial mutations". I think the term "beneficial mutations" is confounding here, as resistance mutations are only beneficial in the presence of antibiotics, and when they appear (in the overnight growth prvevious to plating onto medium supplemented with antibiotics) are mostly deleterious. I would replace the term by "resistance mutations".

The sentence has been rewritten, and it no longer refers to “beneficial mutations”(see lines 231–233).

Line 249. Based on the information in Figure 2, it is not clear why the genotype causing the substitution K88R is mentioned in this sentence, as second-step mutations carrying it do not seem to show any difference in streptomycin resistance. Second-step mutants resistant to kanamycin that carry the substitution K88E show a slight decrease, but the ancestral K88E is also less resistant than other alleles.

Thanks for catching this poorly worded passage. The sentence has been rewritten, and the reference to mutation K88R removed (line 256).

Line 260: I believe the authors mean "4/42, < 10%).

Thank you for catching this mistake. We have corrected it accordingly.

Line 263. I suggest the authors to replace the word "modules" by "functions".

Done.

Line 297. I believe the authors mean "with the exception of K88R".

Thank you spotting this mistake. We have corrected it accordingly.

Line 528. I believe this section is c).

Thanks, we have corrected it accordingly.
