# Supplementary material for: Idiosyncratic evolvability among single-point ribosomal mutants towards multi-aminoglycoside resistance
Source: PLoS Genet. 2025 Aug 25;21(8):e1011832. doi: 10.1371/journal.pgen.1011832 (PMC12416847; doi:10.1371/journal.pgen.1011832)
Supplement: Attachment — Submitted filename: renamed_a085d.docx [file pgen.1011832.s010.docx]

Sánchez-Maroto *et al* investigate the evolvability of *E. coli* MG1655 towards aminoglycoside resistance in a multi-step selection setup. The results reveal epistatic interactions (on fitness and resistance level) between resistance mutations. Additionally, they identified several first-step mutations that pose an evolutionary dead-end towards further resistance selection under the used experimental conditions. The authors discuss their work in a broader context of declining adaptability and diminishing returns of beneficial mutations, and how *rpsL* mutations can act as stepping-stones for resistance towards other antibiotics than streptomycin. The identification of epistatic interactions, and the ‘priming’ of streptomycin resistant mutants towards resistance to other antibiotics is novel and exciting, however there are issues with major claims of the study as well as the experimental execution:

Major

The authors lay out the concept of a declining adaptability and diminishing returns and aim to investigate if antibiotic resistance is underlying these principles. I don’t believe the experimental setup is adequate to answer this question: First, the selective pressures in the study are not consistent, as first step and second step mutations are not selected under the same selective pressure, but rather by (i) streptomycin followed by (ii) different aminoglycosides challenge. Second, not only the selective pressure applied, but also the read-out for ‘fitness’ changes, from streptomycin resistance to resistance (or even mutation frequency) to different antibiotics. Given this, I find an interpretation of the presented results in regards to declining adaptability challenging, and somewhat misleading. Lastly, it is well known that antibiotic resistance genes/mutations often show positive epistatic interactions, even for streptomycin resistance under a constant selective pressure (PMID: 29686259). Thus, the authors premises and motivation such as to “clarify wether declining adaptability patterns … are general phenomena in antibiotic resistance” (Lines 130+131) are confusing and their novelty limited.

The study falls short on any attempt to a mechanistic explanation. It would be interesting to see constructed double mutants of supposably non-viable resistance pathways (a dead-end rpsL mutation with a second step resistance mutation), to determine if resistance can not evolve due to synthetic lethality even in absence of drugs or because no further resistance increase is provided. The authors hint to a potential explanation linked to “increased avoidance of macrophages immune proteins”, “reduced rates of protein oxidative damage” or different translation rates (lines 453-454), but do not perform any experiments towards this end.

The authors discuss their findings in the context of clinical implications, warning that rpsL mutations could prime pathogens for resistance towards other aminoglycosides. To this end, a literature review, or sequence analysis of clinical isolates, would be helpful to understand at what frequency, if at all, the mentioned pathogens (P. aeruginose and A. baumanii (lines 492+493) carry mutations in rpsL, suggesting a contribution to GEN or TOB resstance in the clinics. Is resistance to TOB, KAN, GEN, AMK typically acquired via chromosomal mutations rather than aminoglycoside modifying enzymes? Are the reached resistance levels clinically relevant?

As acknowledged in line 183+184 the five different streptomycin-resistance mutations display a range of fitness effects in absence of the drug. However, the selection was performed without OD (or CFU) normalization. How are mutation frequencies calculated, without determining the starting amount of cells? A different population size will consequently result in different amounts of mutants even without allel specific differences in ‘evolvability’. At a minimum, the authors should repeat some of the selection experiments with OD normalization to show that the observed effects are not affected by different overnight yields.

Materials+Methods section on how mutation frequencies were determined are missing. Assuming the data simply reflects the number of mutants per population, it is not suitable to determine the effect of *rpsL* mutations on genome wide spontaneous mutation rates (unlike fluctuation or mutant accumulation tests).

Line 520-525: The authors state that the rpsL K43N mutant carried an off-target mutation in pflD. To verify the phenotype, two other K43N mutants that did not carry a pflD mutation were tested and behaved similarly to the pflD/rpsL double mutant. Given that mutations in pflD gene have previously been linked to rpsL mutations causing streptomycin resistance (<https://doi.org/10.1021/acsinfecdis.3c00156>), the authors should (i) adress the presence of the off target mutation in the main result section, rather than in mat/met, (ii) whole genome sequence the two K43N clones used for validation to ensure absence of any off-target mutation (especially considering the high-amounts of off-target mutations observed in the second step mutants) and (iii) report the data (susceptibility profile, fitness, mutation frequency) of the two additional clones that is referred to in lines 523+524.

Figure S3: According to Methods, growth rates were determined with a starting OD of 0.01, however, starting OD’s are 0.1 in the figure, suggesting that a background subtraction was not performed. It is also confusing why only the ancestor growth curve starts at time 0, while all others are somewhat delayed. Additionally, the growth curves appear to be linear, but falsely plotted on a logarithmic scale. If correct as plotted, the strains (even the wt) have an unusually long doubling time (>1h?), and a large OD increase during stationary phase. I suspect that during image editing a (fundamental) mistake slipped in, please double check that the presented data is correct and fits the displayed scale.

Figure 3 needs clarification. The colour scheme shows what the mutants were selected on, but it is not fully clear what the MIC values refer to. Assuming that ‘MIC first’ and ‘MIC second / MIC increase’ refer to streptomycin and TOB, AMK, GEN, KAN, respectively, it should also be clarified what the MIC increase refers to. Is it, for example, increase in TOB MIC of the second step mutant towards the first step mutant, or towards the original ancestral strain?

Minor

Figure 1D: AMK doesn’t match Figure 1C – I assume 88E and 88R are mixed up.

Line 511: Should be 100mg/L instead mg/ml.

Figure 1C) Indicate the concentrations (or rather fold X MIC) that the second step mutants were selected on.

Line 163: Authors state that the susceptible strains exhibit lower mutation rates, but later on state that the difference in ‘evolvability’ is not due to differences in mutation rates. Please clarify, or correct if wrong wording.

Line 557: Should be **-**80° freezer instead of 80° freezer.

Figure 2) As stated in line 232, the experiment to determine cross-resistance and collateral sensitivity were performed in triplicate. Please clarify how the results were handled in cases were replicates disagreed

Figure 2) The maximum fold increase is indicated as 10x. Is this really the actual increase in MIC or just the limit of detection? I was under the impression most *rpsL* mutations confer a much higher MIC than 10x wild type, almost limited by streptomycin solubility. Please clarify and add a table with all MIC values for each genotype.

Figure 4) The number of replicates should be stated in the figure legend, and standard deviations should be shown. It would be good to add information, which of the strains carried off-target mutations (which likely act as compensatory mutations) in the figure.

As fairly stated by the authors, the frequent occurrence of off-target mutations in the selected strains is puzzling (lines 376-390). I agree with the arguments, that a stochastic effect is very unlikely, and that the off-target mutations must have appeared after selection as compensate potential fitness deficits. Since strains were grown in liquid to be cryo-preserved as well as for whole genome sequencing, selection for increased growth was not only present during the initial selection, thus I believe the authors comment that “the second mutation became predominant within the colony as it grows on the selective plate” (lines 388+389) is too speculative.
