# Supplementary material for: Idiosyncratic evolvability among single-point ribosomal mutants towards multi-aminoglycoside resistance
Source: PLoS Genet. 2025 Aug 25;21(8):e1011832. doi: 10.1371/journal.pgen.1011832 (PMC12416847; doi:10.1371/journal.pgen.1011832)
Supplement: Attachment — Submitted filename: PGENETICS-D-24-00553_Couce_response_letter.docx [file pgen.1011832.s012.docx]

**Point-by-point response to reviewers for PGENETICS-D-24-00553R1**

**Dear Dr. Crosson,**

**thank you and the reviewers for the careful attention to our paper. We have now addressed the remaining concerns arisen in the last round of review. Overall, we believe the revisions have further strengthened the manuscript, and we are confident it will make a meaningful contribution to the field.**

**In addition to correcting minor typos and ensuring compliance with journal formatting requirements, this revision includes several changes to the text, figures, and tables. Specifically: 1) we now provide an explicit definition of our terminology, addeing three citations from recent high-profile journals to support its common usage; 2) we have improved the captions for Figures 1, S3, and S5; 3) we have expanded the Methods section to elaborate on the selection of first-step mutations and the risk of passenger mutations, leading to the inclusion of a new column in Table 1 and an additional reference; 3) we have added Supplementary Table S4, listing all differences identified in our parental MG1655 strain compared to the GenBank reference. A brief discussion of these differences has been added to the Methods section, along with two more citations.**

**Below, please find a point-by-point response to the reviewers’ comments (in blue), with the original comments shown in black. Changes in the manuscript are highlighted in yellow. A tracked-changes version of the manuscript is also provided, as per journal requirements.**

**On behalf of all authors,**

**Sincerely,**

**Alejandro Couce**

Reviewer #1:

The authors have adequately responded to and implemented my comments and suggestions. The additional experiments improve the manuscript and strengthen the main conclusions.

Thank you for your invaluable feedback. We fully agree that your comments have helped improve the manuscript and strengthen its conclusions.

Minor comments:

The use of the term "dead ends" for some first-step mutants, e.g., in the abstract, remains somewhat misleading and counterintuitive, since the tested case K88E (Figure S2) merely shows a lack of increased evolvability beyond wild type, as also described by the authors in the added paragraph (ll. 210).

Thank you for raising this interesting semantic point. We define a dead-end as a genotype from which further beneficial mutations are not available (i.e., access to more adapted genotypes is not possible via single point mutations). In this sense, the wild-type also qualifies as a dead-end, as it cannot adapt to the second-step antibiotics under the specific conditions tested (and thus adaptation cannot proceed further, much like when a vehicle reaches a literal dead-end).

Another consideration is that the term evolutionary “dead-end” is a conceptual counterpart to the metaphor of a “stepping stone”. In our experience presenting this work, these labels have helped audiences quickly grasp the contrasting roles of different genotypes (in fact, Reviewer 2 also adopted this terminology in their own comments).

Moreover, the term is consistent with usage in recent high-profile papers. For example, Ferrare & Good (2024), *Nat Ecol Evol* define evolutionary dead-ends as genotypes from which “further beneficial mutations are not available”. Similarly, Jagdmann (2022), *PLoS Biol;* Souque (2021) *eLife*, and Lukačišinová (2020), *Nat Commun* use the label “dead-end” to refer to genotypes from which further beneficial mutations are not available.

We have revised the relevant passage (line 210 onwards) to make our definition explicit and to reference this usage in the literature, adding three of the above citations that were not included in the original manuscript. The revised sentence now reads:

"This suggests that these dead-ends result from a failure to enhance evolvability beyond wild-type levels, rather than from direct antagonistic interactions with secondary mutations. Note we use the term “dead-end” to describe genotypes from which no further beneficial mutations are available, consistent with prior literature (5,50,77,78)."

Mutation rate determination (Figure 1, S3 and S5):
• To my understanding, the authors used 3 (first step) or 5 (second step) parallel cultures for plating and mutant counting. The mutation rate was then estimated once. However, the caption of Figure 1 can be interpreted as 5 separate mutation rate estimates and should be rephrased. Notably, 5 or fewer parallel cultures are very few for accurate mutation rate determination in a fluctuation assay.

Thank you for the suggestion. We have amended all three captions to explicitly state that the mutation rate ± 95% confidence interval was “estimated from five (or three) parallel cultures”.

• The mutation rate points look like boxplots and are also labeled as such in the caption of Figure S3. However, they seem to “*represent a single point with the estimated 95% confidence intervals as error* *bars*” in all cases. The appearance should be adjusted and the correct description added in all cases.

Thank you for spotting this oversight, carried over from the earlier version of the figure (that did show boxplots). We have now corrected the caption to explicitly state that values shown are “the mutation rate ± 95% confidence interval, estimated from five parallel replicate cultures”.

Figure 1: Subplots B, C and E all show mutation rates, but the axis limits are different. It would be easier to compare between conditions based on one plot range.

Thank you for this suggestion. We did carefully consider axis scaling to balance comparability and readability. Due to the wide range of values—especially the 95% confidence intervals—using a single y-axis range resulted in overly compressed plots that hindered clarity. The compromise we adopted is reflected in Figure 1C: panels are grouped by value range, with the upper row spanning 10⁻⁹ to 10⁻⁶ and the lower row spanning 10⁻⁸ to 10⁻⁵. Moreover, we ensured that all panels cover exactly three orders of magnitude, facilitating comparison across the upper and lower groups. Finally, note that Figure 1E compiles the values from Figure 1C into a single plot, allowing direct comparison across all treatments. We now explicitly clarify this design choice in the figure caption:

“Note that panels are arranged in upper and lower rows based on shared value ranges, but all span exactly three orders of magnitude to facilitate comparison; see (E) for direct comparison of values in a single plot.”

l. 308: The reference to Figure S3 should now be changed to S4 due to the addition of the mutation rate determination. Same in the caption of Figure 4 (l. 998).

Thanks for catching this. We've updated the relevant passages to reflect that former Figure S3 is now S4.

Supplementary Information l. 106: The CRISPR/Cas9 primer table should be Table S3.

Corrected. We appreciate again your attention to detail.

Reviewer #3:

I appreciate the efforts made by the authors of the manuscript entitled "Idiosyncratic evolvability among single-point ribosomal mutants towards multi-aminoglycoside resistance", by Sánchez-Maroto et al. to address the concerns expressed by me and other two reviewers. The authors performed new experiments and significantly edited the text of the manuscript, and these efforts should be commended. A fraction of my concerns are completely or partially mitigated by the data and discussions in the revised version of the manuscript. Unfortunately, there are others that, in my opinion, still require further action. These concerns are discussed below, maintaining the nomenclature of my previous comment.

We appreciate your thoughtful evaluation and are glad to hear that several of your concerns have already been satisfactorily addressed. We respond to the remaining points below.

MAJOR CONCERN 1. In my opinion, not calculating the average number of generations in the new experiment with fosfomycin was a lost opportunity, as could have allowed the authors to experimentally estimate mutation rates, besides the mathematical inference shown in the revised version of the manuscript. However, such inference shows no significant differences in mutation rates, and data used for it was obtained in a new independent experiment selecting for alleles that in principle do not interact epistatically with streptoymcin resistant alleles. Thus, my major concern 1 can be considered as mitigated.

Thank you for this observation. we're pleased that the new data and analyses were sufficient to mitigate your concerns.

MAJOR CONCERNS 2 AND 3. The low probability of sampling preexisting mutants upon 10−4 dilution is based on the assumption that the frequency of newly arisen mutants in the bacterial population remains stable since the appearance of the mutation until the moment of sampling. However, if these newly arisen mutations are neutral or beneficial (for instance, if they cause positive epistasis with other polymorphisms present in the parental background, or if they appear in a genetic background that already carried a beneficial mutation), frequency of newly appeared mutants can be much higher than the estimated ~10−9 at the time of sampling, especially if they appeared early in the growth of the previous liquid culture. Indeed, the fact the the authors found the same mutation in pflD in all derivatives of the K43N background proves that the purpose of eliminating "any mutants that may have arisen in the initial overnight culture" was defeated, and that this experimental approach does not prevent selection of passenger mutations. The effect of mutations other than the alleles conferring antibiotic resistance can only be ruled out if multiple initial cultures are run in parallel, growth steps are minimized, results from these independent initial cultures are compared, and clones carrying additional mutations are excluded from the analysis. This would require essentially repeating most of the experiments that constitute the core of this work, and I do not think requesting that would be fair to the authors at this point. An alternative way to mitigate these concerns would thus be to discuss in the main text of the manuscript the limitations of the approach selected, as well as the point discussed below.

Moreover, it is not clear if other mutations, even if they did not appear in all the replicates from the same genetic background, were detected after performing genome sequencing of the 18 strains derived from first-step mutants. The presence of any specific allele in more than one replicate derived from the same streptomycin resistant background would indicate its appearance during selection of first-step mutants (unless one assumes the unlikely scenario of simultaneous appearance of the very same allele in independent cultures). I thus think the full list of mutations found in the sequencing of the 18 isolates should be available, and appearance of parallel mutations in replicates from the same streptomycin resistant background discussed in the main text of the manuscript.

Thank you for insisting on this point. First, let us clarify: the purpose of the standard dilution step is not to eliminate passenger mutations, but to minimize the chance of sampling pre-existing resistance mutations (with resistance mutation frequencies on the order of 10^⁻9^, this probability drops to ~0.001%). We have now stated this explicitly in the relevant passage and corrected the estimate (lines 540–543 in the tracked changes version).

Second, we agree that passenger mutations can occasionally hitchhike with resistance mutations selected later on. Given a genome-wide mutation rate of ~10⁻³ (Lee et al., 2012 *PNAS*), the expected frequency of co-occurring passengers with resistance mutations is ~10⁻^12^, so in a typical 10⁹-cell population the probability of selecting a double mutant is on the order of 0.1%. This figure is still low, but fairly within the realm of what can happen during standard laboratory procedures. Moreover, as you point out, this estimate reflects an average across many cultures and does not account for early-arising mutations (i.e., “jackpot” events), which could raise the probability several-fold in a given population (Couce 2016, *Genetics*).

How could we then guard against the possibility of sampling co-occurring passenger and resistance mutations? Your suggestion of reducing growth time to limit mutation accumulation is not feasible, as smaller populations would rarely generate the very resistance mutations we need. In retrospect, the only effective safeguard would have been to whole-genome sequence the first-step mutants before proceeding to the second-step – but at the cost of a slower turnaround.

Ultimately, what matters is that we sequenced all 18 second-step derivatives and found no additional mutations beyond the pflD mutation, which was present in all descendants of the K43N mutant. Your comment made us realize that the absence of other potential passenger mutations was not clearly conveyed. We have now added pflD to Table 1 and clarified in the legend that it includes all mutations detected among second-step derivatives. We have also expanded the relevant Methods section to elaborate on the limitations discussed above. An additional reference has been added (Lee et al., 2012 *PNAS*). Finally, we have moved the discussion of the *pflD* mutation occuring in all derivatives of the K43N background from the Methods section to the Results section.

MAJOR CONCERN 4. I still think the term "evolutionary dead end" is misleading. Even more so in light of the results obtained by the authors in response to th reviewer's comments (shown in the new Supplementary Figure S2), which demonstrate that even in the most extreme case, evolvability of the so-called evolutionary dead-ends is not lower than that of the wild type (actually, is higher for one antibiotic). Consideration of the notions "evolutionary stepping-stone" and "evolutionary dead-end" as absolute categories is oversimplistic. The fact that certain mutations conferring resistance to streptomycin can open additional evolutionary pathways towards resistance to other aminoglycoside can certainly be considered an evolutionary stepping-stone. But calling other mutations "evolutionary dead-end" suggests that they cause the opposite effect; that is, that they reduce evolvability when, in reality, they simply do not affect evolvability at all. In my opinion the confusion will be eliminated if the authors simply state that some mutations constitute evolutionary stepping-stones and others do not.

Thank you for insisting on this semantic point. We define a 'dead-end' as a genotype that, in our selective context, has no access to further adaptive mutations. This definition is consistent with usage in recent high-profile papers. For example, Ferrare & Good (2024), *Nat Ecol Evol* define evolutionary dead-ends as genotypes from which “further beneficial mutations are not available”. Similarly, Jagdmann (2022), *PLoS Biol;* Souque (2021) *eLife*, and Lukačišinová (2020), *Nat Commun* use the label “dead-end” to refer to genotypes from which further beneficial mutations are not available. In this sense, the wild-type genotype is also a dead-end, as it consistently fails to generate second-step mutants (and thus adaptation cannot proceed further, much like when a vehicle reaches a literal dead-end).

Moreover, in our experience presenting this work, the metaphorical labels “dead-end” and “stepping-stone” have proven helpful in conveying the contrasting roles of different genotypes. In fact, Reviewer 2 adopted this terminology in their own comments. That said, we acknowledge that metaphors can be interpreted differently by readers from various fields, so we have now revised the relevant passage (line 210 onwards) to make our definition explicit and to point at examples of this very same usage in the literature (resulting on the addtion three of the above citations that were not included in the original manuscript).

MINOR CONCERN A. Table 1 and Figure S2 in PMID: 33830249 indeed show that E. coli streptomycin resistant mutants carrying the K43N substitution in rpsL show no apparent difference in cell shape and frecuency of DNA breaks (and therefore SOS induction). However, Figure S1 shows a strong induction of the SOS response in the strain carrying the K88E substitution. Since activation of the SOS response causes inhibition of cell division and thus cell elongation, this mutant is expected to be strongly affected in cell shape. Thus, I still think competitive fitness of the resistant genotypes under study against a wild-type reference would be a more accurate representation of fitness than growth curves. However, since this would involve a significant amount of work at this stage of the publication process, if other reviewers and the editor accept growth curves, I'd be happy to go with the majority.

We agree that competition assays might have provided a interesting measure of fitness, but implementing them at this stage would require unnecessary additional work (as growth curves offer an acceptable approach to support our main findings). In any case, we're pleased to hear that you are satisfied.

MINOR CONCERN B. Spontaneous E. coli resistant mutants carrying the strA60 allele, causing the amino acid substitution K43R, have been repeatedly isolated by multiple laboratories in the last 50 years (e. g. PMID: 5326090; PMID: 4920897; PMID: 4560854; PMID: 19629166). It is thus not an uncommon allele to appear in E. coli. According to section b of materials and methods (lines 533-540 in the revised version of the manuscript), a unique bacterial culture was grown overnight and then multiple dilutions of the very same culture were grown overnight again, prior to plating onto LB agar supplemented with streptomycin, to select for mutants. In that scenario of a single biological replicate diluted into several replicates, biases towards particular alleles appearing early in the initial overnight cultures are expected, and therefore the absence of certain substitutions, including K43R, is not that much surprising. However, in their response, the authors mention the possibility of epistatic interactions with other polymorphisms as possible factors causing the lack of appearance of this allele in their experiments. Since the genotype of the ancestral strain used (or a strain name and its corresponding reference) is not provided in the initial nor in the revised version of the manuscript, it is possible this interpretation by the authors is correct and other polymorphisms are hampering the appearance of the allele causing the K43R substitution. In order to rule out (or confirm) this hypothesis, and considering that the ancestral genetic background is paramount in this type of studies, a clear description of the strain used is necessary and, if any mutation potentially explaining the absence of the allele causing the K43R substitution (or any other allele causing streptomycin resistance) is present in ancestral strain, this must be discussed in the main text of the manuscript.

Thank you for your suggestions. While we did include the name of the ancestral strain in the first sentence of the Methods section (*Escherichia coli* K-12 strain MG1655), we agree that providing the full genotype is helpful for future reference. It is well known that the same strain can differ slightly across laboratories due to mutations accumulated through repeated cycles of cultivation, streaking, and storage (e.g., in the case of MG1655, PMID: 26647077, 25741363, 31776414).

Accordingly, we have now added a new Supplementary Table S4 listing all differences identified in our parental MG1655 strain compared to the GenBank reference (U00096.3). As is typical, we found five differences, all previously reported in other MG1655 derivatives (PMID: 26647077; 25741363). Two map to pseudogenes, two to intergenic regions, and one corresponds to the deletion of a mobile element (IS5). None have a clear functional connection to streptomycin resistance, and the variant closest to the *rpsL* locus is located over 0.5 Mbp away—a considerable genomic distance (~10% of the genome). This brief discussion has also been added to the corresponding Methods section, including two new references (PMID: 26647077; 25741363).
