# Supplementary material for: Idiosyncratic evolvability among single-point ribosomal mutants towards multi-aminoglycoside resistance
Source: PLoS Genet. 2025 Aug 25;21(8):e1011832. doi: 10.1371/journal.pgen.1011832 (PMC12416847; doi:10.1371/journal.pgen.1011832)
Supplement: Attachment — Submitted filename: PGENETICS-D-24-00553R2_Couce_response_letter.docx [file pgen.1011832.s013.docx]

**Point-by-point response to reviewers for PGENETICS-D-24-00553R2**

**Dear Dr. Crosson,**

**thank you and the reviewers once again for the careful attention to our paper. We have now addressed the final remaining concern raised by Reviewer #3. As detailed in our rebuttal, the reviewer misinterprets the common usage of the term “evolutionary dead end”, assuming it refers to genotypes that merely reduce evolvability relative to the ancestral genotype. Neither we nor the authors of the cited studies use the term this way. Instead, we apply it to a genotype that cannot adapt any further, while other alternative genotypes remain evolvable under the same selective conditions. That is, the term “dead end” is used** in relation to other **adapting** **variants, not to the ancestor. To ensure this is entirely clear to all readers, we have expanded the relevant paragraph in the manuscript to clarify the standard usage of the term.**

**As noted in your letter, all other reviewer comments have been addressed to their satisfaction, including those requiring substantial new experiments, analyses, and figures. After more than one year and three rounds of revision, we are grateful for the constructive nature of this review process, which has clearly strengthened the manuscript. Below, please find our point-by-point response (in blue) to the reviewers’ comments (in black). Changes in the manuscript are highlighted in yellow. A tracked-changes version of the manuscript is also provided, as per journal requirements.**

**On behalf of all authors,**

**Sincerely,**

**Alejandro Couce**

Reviewer #1:

The authors have addressed my remaining comments convincingly. I support the publication of the manuscript.

Thank you again for your very useful feedback.

Reviewer #3:

The authors of the manuscript entitled "Idiosyncratic evolvability among single-point ribosomal mutants towards multi-aminoglycoside resistance", have made a commendable effort to address my remaining concerns. Further changes in the text of the manuscript mitigate major concerns 1, 2 and 3 and minor concerns A and B. However, major concern 4 still remains.

I understand the intention of the authors when defining "evolutionary dead end", and I appreciate their efforts to clarify their particular definition. Unfortunately, I think this nomenclature is not only semantic, but conceptually incorrect. The authors seem to conceive a binary separation between "stepping-stone" and "dead end". That is, any mutation increasing evolvability can be considered a "stepping stone" mutation, and any mutation not increasing evolvability can be considered an evolutionary "dead end". This bipolar nomenclature does not allow to distinguish between mutations that do not affect evolvability and mutations that decrease evolvability; it thus bundles together mutations that are neutral to the phenotype under study (evolvability) with those negatively affecting it. In my opinion this significant biological and evolutionary difference cannot be ignored, making the nomenclature proposed by the authors misleading.

Regarding the literature used by the authors to discuss this point (PMID: 39261599, PMID: 36170241, PMID: 33634790, and PMID: 32561723), I fear does not really support their perspective. For instance, the paragraph where Ferrare and Good (PMID: 39261599) disscuss the concept of evlutionary "dead end" reads as follows: "The opposite behavior occurs when short-term fitness benefits are linked to long-term reductions in evolvability. In this case, modifiers that would be strongly disfavored on their own [ fix⁡(( , )→( ′ , ′ ))≪⁢1] will generally amplify the relative contribution of a direct fitness benefit (Fig. 3B,C). A striking example of this effect occurs in the extreme case of an evolutionary “dead end”, where further beneficial mutations are not available." Thus, Ferrare and Good define evolutionary "dead ends"as a specific case occurring when "short-term fitness benefits are linked to long-term reductions in evolvability".

Similarly, Jagdmann et al. (PMID: 36170241) discuss the concept of evolutionary "dead end" in the context of a deletion in the secuence encoding the TetR repressor, which prevents selection of resistance to tygecycline mediated by gene amplification. In that scenario, strains carrying the deletion clearly suffer a reduction in evolvability compared to the strains not carrying it. Finally, Souque et al. (PMID: 33634790) use the term "evolutionary dead end" referring to the context of a particular experiment (which required evolving strains to survive to increasing concentrations of gentamicin), while discussing the effect of the tandem duplication of an antibiotic resistance gene (aadB) in an artificial three-cassette integron; the absence of tandem duplication at concentrations beyond 4X the MIC made the authors infer that it only conferred a small increase in gentamicin resistance, and did not allow to acquire additional mutations that further increase resistance. Thus, the phenomenon described by Souque el al. also involves a reduction in evolvability. Lukačišinová et al. (PMID: 32561723) literally study and genes that, when deleted, reduce evolvability of genotypes carrying these deletions with respect to the ancestral reference strain. This work thus also agrees with the necessity of a reduction in evolvability in order to define an evolutionary "dead end".

In conclusion, according to the literature proposed by the authors, genotypes unable to generate second-step mutants could only be considered "evolutionary dead-ends" if they suffered a reduction in evolvability with respect to their ancestral reference. Streptomycin-resistant mutants did not underwent any reduction in evolvability compared to wild-type bacteria. And wild-type bacteria did not suffer decreased evolvability with respect to itself. These genotypes simply did not benefit from the increase in evolvability caused by the acquisition of "stepping-stone" mutations. Thus, genotypes not increasing evolvability cannot be conssidered "evolutionary dead ends". I encourage the authors to define the phenotypes observed precisely, avoiding the use of terms that cannot be specifically adscribed to their observations, such as "evolutionary dead end".

Thanks for insisting on this point. Our usage is conceptually sound and consistent with the literature, and we are happy to clarify once more this misunderstanding. You state that we define a “dead-end” as “any mutation not increasing evolvability”. This is fundamentally incorrect: neither we nor the authors of the referenced studies use the term to describe mutations that merely fail to enhance evolvability. Rather, all of us reserve it for the extreme case in which evolvability is curtailed to zero; that is, when “further beneficial mutations are not available”. Note this is the exact wording we use, and it matches exactly that of Ferrare & Good (2025, Nat. Ecol. Evol.), who define an evolutionary “dead end” as a case where “further beneficial mutations are not available”. This usage follows the standard figurative meaning of “dead end” in plain English: “a position, situation, or course of action that leads to nothing further” (Merriam-Webster), and is commonly applied in evolutionary biology to genotypes from which further adaptation is no longer possible.

We believe your misunderstanding stems from not recognizing that the label “evolutionary dead-end” is applied relative to alternative evolutionary paths, not with respect to the ancestral genotype. In our work, the wild-type cannot survive the streptomycin lethal dose at all (the first adaptive step), so the only relevant comparison is among the surviving first-step genotypes. Therefore, it makes no sense to entertain any philosophical debate about whether an evolved mutant has more or less evolvability than the wild type, which goes extinct at the outset. The references we cited use the term in exactly the same way, to describe evolved genotypes that terminate the line of adaptive improvement under specific selective conditions and in relation to other alterantive variants. For the record:

- Ferrare & Good (2025, Nat. Ecol. Evol.): the authors discussed "dead-ends" (a genotype from which “further beneficial mutations are not available”) as an extreme case in the context of competing lineages with or without evolvability-enhancing mutations. In other words, dead-ends are determined by their adaptive stasis while others continue adapting, not by a decrease in evolvability compared to the wild type.

- Jagdmann et al. (2022, PLoS Biol): the authors characterize a specific *tetR* mutation as “an evolutionary dead-end that precludes the evolution towards high-level tigecycline resistance”. This genotype cannot achieve high-level resistance, unlike other tetR variants that do so via tandem amplifications. Much like in our case, the comparison is among alternative mutant genotypes within a stepwise selection framework; never between mutants and the wild type.

- Souqué et al. (2021, eLife): the authors refer to one integron configuration as a “dead end under strong selection for elevated resistance”. In their system, various initial genotypes (integron cassette orders) lead to different evolutionary outcomes under gentamicin selection. One particular arrangement confers only a slight benefit at low drug levels but ultimately cannot evolve further under high gentamicin pressure, becoming an evolutionary dead-end. Again, this usage is clearly relative to other genotypes that do permit further adaptation. The wild-type plays no role in this comparison as it is eliminated as soon as the experiment starts, just as in our work.

- Lukačišinová et al. (2020, Nat. Commun.): here the authors use the term "dead-end" in the introduction: “More sustainable drug treatments … require novel strategies that anticipate the evolutionary potential of pathogens and funnel them toward less evolvable genotypes or evolutionary dead ends”. Here, “dead ends” are presented as the extreme case of minimal evolvability, where a pathogen’s adaptive potential is virtually zero. They refer to interventions designed to steer evolution toward specific trajectories among the various adaptive routes available to a pathogen. Again, there is no implication that a “dead-end” has lower evolvability than the wild type, only that it has none relative to other alternative evolutionary paths.

In any case, to ensure that the main text makes this definition even clearer, we have expanded the relevant passage as follows:

"Note we use the term “dead-end” to describe genotypes from which no further beneficial mutations are available, thus terminating the line of adaptive evolution. Crucially, this term is defined within the context of our selective regime (i.e., lethal streptomycin followed by secondary aminoglycosides) and is therefore used in relation to alternative adapting variants—not the ancestor, which is eliminated at the outset. This usage is consistent with prior literature, which applies the term to the extreme case of genotypes that cannot adapt any further, while other alternative genotypes remain evolvable under the same selective conditions (5,50,77,78)."

We trust that this clarification addresses your concern once and for all, demonstrating that our nomenclature is both precise and consistent with established usage.
